# Getting Histoplasmosis on the Map of International Recommendations for Patients with Advanced HIV Disease

**DOI:** 10.3390/jof5030080

**Published:** 2019-09-02

**Authors:** Felix Bongomin, Richard Kwizera, David W. Denning

**Affiliations:** 1 Department of Medical Microbiology & Immunology, Faculty of Medicine, Gulu University, Gulu P.O. Box 166, Uganda; 2 Global Action Fund for Fungal Infections, Rue Le Corbusier 12, 1208 Geneva, Switzerland; 3 Infectious Diseases Institute, College of Health Sciences, Makerere University, Kampala P.O. Box 22418, Uganda; 4 Makerere University Lung Institute, College of Health Sciences, Makerere University, Kampala P.O. Box 7749, Uganda; 5 The National Aspergillosis Centre, Wythenshawe Hospital, The University of Manchester, Manchester Academic Health Science Centre, Manchester M23 9LT, UK

**Keywords:** histoplasmosis, HIV, *Histoplasma* antigen tests, itraconazole, amphotericin B

## Abstract

Progressive disseminated histoplasmosis, caused by *H. capsulatum*, is a life-threatening illness and is an AIDS-defining opportunistic infection. It is neglected, worryingly under-diagnosed, and often misdiagnosed as cancer or tuberculosis with fatal consequences. Globally, over 100,000 cases of disseminated histoplasmosis have been estimated. In 2017, the World Health Organization (WHO) noted that disseminated histoplasmosis is a significant cause of mortality in AIDS patients. Through the rigorous efforts of the Global Action Fund for Fungal Infections (GAFFI) and partners, in 2019, the *Histoplasma* antigen test was included on the 2nd Edition of the WHO List of Essential Diagnostics. The drugs used in the treatment of histoplasmosis (amphotericin B and itraconazole) are on the WHO Essential Medicine List. The *Manaus Declaration* on histoplasmosis in the Americas and the Caribbean, where histoplasmosis kills more people with HIV than tuberculosis, advocates for universal access to rapid testing for histoplasmosis and availability of essential drugs for the treatment of histoplasmosis in every country by 2025. Hyperendemic areas are present in the Americas, Caribbean, Southeast Asia, and Latin America. In conclusion, histoplasmosis remains an important clinical and public health problem. To reduce HIV-associated mortality, disseminated histoplasmosis must be addressed through advocacy, increased awareness, and universal access to essential diagnostics and antifungal agents.

## 1. Introduction

Fungal infections are still a major cause of opportunistic infections among people living with human immunodeficiency virus (HIV), especially in low and middle-income countries [1]. Among them is disseminated histoplasmosis, which is responsible for numerous deaths in this population [2,3]. Histoplasmosis refers to a spectrum of clinical syndromes, caused by the ubiquitous and thermally dimorphic fungi of the genus *Histoplasma*, of which two varieties are known to infect humans, that is, *Histoplasma capsulatum* var. *capsulatum* (*H. capsulatum*) and *Histoplasma capsulatum* var. *duboisii* (*H. duboisii*). *H. capsulatum* is more common, with a worldwide distribution, while *H. duboisii* is endemic on the African continent [4,5]. *Histoplasma* is transmitted by way of the respiratory tract, but once inhaled into the alveoli, several disease processes may follow, including acute pulmonary histoplasmosis, chronic cavitary pulmonary histoplasmosis (primarily in those with chronic obstructive pulmonary disease), and both acute and subacute disseminated histoplasmosis in the immunocompromised host [6,7]. A vast majority of immunocompetent individuals are asymptomatic and are only identified through a reactive histoplasmin skin test [8]. In advanced HIV infection or acquired immunodeficiency syndrome (AIDS), the organism readily spreads throughout the body, causing a wide spectrum of manifestations; notably, fever, hepatosplenomegaly, lymphadenopathy, gastrointestinal symptoms, pneumonia, skin lesions, and pancytopenia [2,9,10]. This communication aimed to overview the epidemiology, diagnosis, and treatment of histoplasmosis, and to highlight the key steps and advocacy undertaken to tackle the morbidity and mortality associated with the disease. 

## 2. Risk Factors for Histoplasmosis

*Histoplasma* is a primary pathogen capable of causing an asymptomatic infection or a self-limiting pulmonary disease in immunocompetent hosts, as well as causing an acute or subacute progressive disseminated disease in patients with significant immune suppression following a) acquisition of a primary infection or b) reactivation of latent infection [6,7]. Progressive disseminated histoplasmosis, primarily caused by *H. capsulatum* is a life-threatening illness and is an AIDS-defining opportunistic infection, included on the World Health Organization (WHO) stage 4/US Centers for Disease Control (CDC) category C events since 1987 [1,3]. Disseminated disease mainly occurs in severely immunocompromised HIV-infected patients with CD4 counts less than 50 cells per microliter, though disseminated disease can also occur in those with CD4 counts less than 100 cells per microliter. Other risk factors for disseminated histoplasmosis are idiopathic CD4 lymphopaenia, adult immunodeficiency syndrome, and hyper IgE (Job’s) syndrome [7]. 

## 3. Burden of Histoplasmosis

Endemic areas for histoplasmosis include the Americas, Africa, and Southeast Asia, including southern China and India [1,5,11,12,13]. Regions of endemicity have expanded in the last 10 years with more southerly parts of Argentina [14] and more northerly parts of the United States [13]. Localities not normally associated with histoplasmosis may yield occasional cases because of transfer of infected soil. 

Progressive disseminated histoplasmosis is an increasingly commonly recognised cause of infection in patients with advanced HIV disease from areas endemic for histoplasmosis [3,15,16]. The true burden of HIV-associated disseminated histoplasmosis remains unknown since it is not a notifiable disease [17]. Globally, about half a million people get infected with *Histoplasma* infection every year. However, approximately 100,000 people develop disseminated disease [18], with mortality rates, if treated, ranging between 30 to 50% [3,19,20], and 100% if not. 

Despite the global expansion of antiretroviral therapy, HIV-associated disseminated histoplasmosis remains a significant public health issue with low-income and high-income countries exhibiting a disproportionate morbidity and mortality [2,16,21]. Large and small outbreaks have been attributed to histoplasmosis, but most infections are sporadic [4]. Histoplasmosis is a major killer of HIV-infected patients in South and Central America [2,22]. In 2012, about 6710 to 15,657 cases of symptomatic HIV-associated histoplasmosis were estimated in Latin America, with areas such as Central America, the northern most part of South America, and Argentina having a prevalence above 30% and incidence greater than 1.5 cases per 100 people living with HIV, resulting in about 671 to 9394 deaths related to histoplasmosis, compared with 5062 deaths related to tuberculosis reported in the region [23].

A more recent systematic review from Brazil confirmed HIV as the most common underlying disease in disseminated histoplasmosis, with mortality as high as 33.1% of the over 3500 cases published by December, 2018 [20]. Still in South America, the Global Action Fund for Fungal Infections (GAFFI) Guatemala pilot project providing rapid testing for opportunistic infections in AIDS has demonstrated that over 60% of life-threatening illnesses among AIDS patients in Guatemala are fungal, mostly histoplasmosis.

A very recent systematic literature review of cases of all disease forms of histoplasmosis in Southeast Asia, not including the Indian sub-continent, found a total of 407 cases [24]. Most cases (255 (63%)) were disseminated histoplasmosis and 177 (43%) cases were HIV-associated [24]. The highest burden of histoplasmosis was noted in Myanmar, Bali, and Surabaya in Indonesia, Ho Chi Minh City in Vietnam, southern Thailand, and north Luzon in the Philippines.

In the East African region, a study from Northern Tanzania retrospectively identified 9 (0.9%) cases of probable histoplasmosis among 970 febrile inpatients, 6 (66.7%) of whom were HIV-infected and 7 (77.8%) who were clinically misdiagnosed as tuberculosis or bacterial pneumonia [25]. In Uganda, 1.3% (2/151) of HIV-infected persons with suspected meningitis were serum *Histoplasma* IgG-positive [26]. 

## 4. Diagnosis of Histoplasmosis

Disseminated histoplasmosis often resembles and can be misdiagnosed as pulmonary tuberculosis and is a major cause of death among HIV-infected patients [16]. The presence of skin lesions, which can be biopsied, is helpful if present (but they are similar to those of *Talaromyces marneffei* and *Cryptococcus neoformans* infections, the so called *Molluscum contagiosum*-like skin lesions). Gastrointestinal symptoms are often prominent in disseminated histoplasmosis, unlike in tuberculosis. Pancytopenia is more profound than in other patients with advanced HIV disease. 

A definite diagnosis of histoplasmosis is based on the isolation of the organisms in fungal culture [27]. Direct microscopy of wet preparations of clinical specimens is not a suitable method of diagnosis of histoplasmosis; and stained smears are preferred [28,29]. Besides, direct examination to identify dimorphic fungi requires trained mycologists, yet access to laboratories for culture in low- and middle-income countries is limited. Serological tests for *H. capsulatum* antibodies are most useful in the diagnosis of sub-acute pulmonary disease, chronic pulmonary disease, granulomatous mediastinitis, and pericarditis [2,30]. In the immunocompromised person, *Histoplasma* polysaccharide antigen detection tests allow rapid diagnosis of disseminated histoplasmosis in urine, serum, bronchoalveolar lavage (BAL) [31,32], and cerebrospinal fluid (CSF) samples before positive cultures can be identified [33]. Antigen concentration is greatest in urine and can be used to monitor response to antifungal therapy and to identify relapsing patients [34]. However, cross-reactivity between the agents of other endemic mycoses should be considered in interpreting the results of the *Histoplasma* antigen tests [6,7].

In AIDS patients with disseminated disease, *Histoplasma* antigen has been detected in the urine of 95–100% and in the serum of 80% of the patients [7,30,35,36]. The availability of a simple, rapid method to detect *H. capsulatum* infection in low- and middle-income countries would dramatically decrease the time to diagnosis and treatment, and deaths in patients with AIDS-related disseminated histoplasmosis [31,37]. At present, however, only one ELISA test is commercially available [38], and none of the lateral flow assays (LFAs) in development have been commercialised, although this is likely in 2019. 

Diagnosis of histoplasmosis can be achieved in about 40% of cases by careful examination of a stained blood film, compared to over 90% of cases when bone marrow is examined [7]. It should be noted that the sensitivity of blood smear is dependent on the degree of disseminated disease; thus, diagnosis of histoplasmosis by this method should not be done in early stages or low fungal burdens. On the other hand, antigen detection in serum or urine has a sensitivity of about 70–90% and molecular methods on blood are >95% sensitive [7,30]. Improved detection rates of histoplasmosis using either antigen detection assays or molecular methods have been shown to reduce early mortality [3]. A systematic review and meta-analysis of the diagnostic accuracy of *Histoplasma* antigen detection tests concluded that these tests are promising tools to improve detection of histoplasmosis, and if made widely available and accessible, would ultimately reduce the burden of histoplasmosis mortality in patients with advanced HIV disease [31].

## 5. Treatment of Histoplasmosis

HIV-associated disseminated histoplasmosis always requires antifungal treatment in addition to effective antiretroviral therapy. Histoplasmosis is a treatable condition and antifungal treatment is highly effective, even in immunocompromised patients, such as those with advanced HIV disease [7]. The choice of antifungal treatment for disseminated histoplasmosis depends on the clinical severity of the disease, with guidelines recommending 1–2 weeks of intravenous amphotericin B for severe and moderate-to-severe disseminated histoplasmosis, followed by itraconazole for a total treatment duration of 1 year [4,39]. Oral itraconazole alone is used for the treatment of mild-to-moderate disease. Liposomal amphotericin B is superior to conventional amphotericin B, both in terms of response rate and survival benefit [4,39]. It is not clear if anti-retroviral therapy should be or should not be started immediately on treatment of histoplasmosis, but it probably should be, based on experience with *Talaromyces marneffei*.

## 6. Efforts Taken to Advocate for the Fight against Histoplasmosis

Recognition of disseminated histoplasmosis as an AIDS-defining opportunistic infection by the WHO and the CDC in 1987 was an important indicator of a sinister illness. Over the past few decades, there has been a significant development in the epidemiology, diagnosis, and treatment of HIV-associated disseminated histoplasmosis. In 2017, the WHO recognized histoplasmosis as a significant opportunistic infection and a major cause of death in patients with advanced HIV disease, especially in the hyperendemic areas [40]. In addition, the WHO added itraconazole on the “2017 Model List of Essential Medicines” for adults (EML) for management of selected fungal infections, including histoplasmosis [41]. This gives hope that resource-constrained nations can access itraconazole for the management of histoplasmosis, as it is off patent and there are many generic suppliers.

In April 2018, GAFFI organised a conference in collaboration with multiple partners to gather experts and review evidence on available in vitro diagnostics for opportunistic infections (including histoplasmosis) in advanced HIV disease for inclusion on the WHO Model List of Essential In Vitro Diagnostics (EDL). The panel of experts very strongly recommended *Histoplasma* antigen detection as an essential diagnostic in endemic areas, or in non-endemic areas as a reference test for imported cases of histoplasmosis [37]. In 2019, with studies showing convincing evidence on the usefulness of *Histoplasma* antigen tests, the WHO included *Histoplasma* antigen tests on the 2nd Edition of the EDL [42]. 

In March 2019, the *Manaus Declaration* on histoplasmosis in the Americas and the Caribbean launched the “100 by 2025” target. It established that, by 2025, every country should have access to rapid testing for histoplasmosis (antigen or polymerase chain reaction/molecular), and availability of itraconazole and both conventional (deoxycholate) and lipid formulations of amphotericin B in the public sector. This needs extension to other hyperendemic areas.

In conclusion, despite the global roll out of antiretroviral therapy, histoplasmosis remains an important clinical and public health challenge in low- and middle-income countries, causing life-threatening disease among HIV-infected persons. Disseminated histoplasmosis should always be considered in the evaluation of an acutely ill HIV-infected patient. Improved clinician awareness will reduce the under-/misdiagnosis of histoplasmosis, and universal access to rapid and accurate diagnostic tools and essential antifungals would greatly reduce time to diagnosis, improving treatment response, and survival.
